# The Clinical Utility and Plausibility of Oxidative and Antioxidant Variables in Chronic and End-Stage Kidney Disease: A Review of the Literature

**DOI:** 10.3390/ijms26073376

**Published:** 2025-04-04

**Authors:** Ariti Tsinari, Stefanos Roumeliotis, Ioannis E. Neofytou, Garyfallia Varouktsi, Andrej Veljkovic, Aikaterini Stamou, Konstantinos Leivaditis, Vassilios Liakopoulos

**Affiliations:** 1 2nd Department of Nephrology, AHEPA Hospital, Medical School, Aristotle University of Thessaloniki, 54636 Thessaloniki, Greece; arititsi@gmail.com (A.T.); john_neofytou_@hotmail.com (I.E.N.); varoukts@uth.gr (G.V.); katerina_stms@yahoo.gr (A.S.); konleiv@windowslive.com (K.L.); liakopul@otenet.gr (V.L.); 2 Department of Biochemistry, Faculty of Medicine, University of Niš, 18000 Niš, Serbia; veljkovicandrej@yahoo.com

**Keywords:** antioxidants, cardiovascular disease, chronic kidney disease, hemodialysis, lipid peroxidation, malondialdehyde, N-acetylcysteine, peritoneal dialysis, protein oxidation, vitamin E

## Abstract

Oxidative stress (OS) is caused by an imbalance between the production of reactive oxygen species (ROS) in cells and tissues and the ability of the biological system to detoxify these products. In chronic kidney disease (CKD), OS contributes to deterioration of kidney function and disease progression. In patients with end-stage kidney disease undergoing hemodialysis or peritoneal dialysis, OS is further increased and associated with adverse clinical outcomes, including deterioration and subsequent loss of residual renal function, atherosclerosis, hypertension, cardiovascular disease and death. However, currently, there is no consensus or guidelines for the diagnosis and treatment of OS in these patients. Herein, we aim to present the existing data regarding biomarkers of OS, pro-oxidants (oxidized albumin, advanced oxidation protein products, xanthine oxidase/dehydrogenase, nitrite/nitrate, malondialdehyde) and antioxidants (superoxide dismutase, catalase, vitamin E, total antioxidant capacity, N-acetylcysteine) that are most clinically relevant and have been more extensively studied in patients with chronic kidney disease, aiming to provide a clearer understanding of this complex area.

## 1. Introduction

ROS are highly reactive molecules and free radicals, containing at least one atom of oxygen, that are produced during the normal metabolism of oxygen inside the mitochondrial matrix. Within normal ranges, ROS are intrinsic to cell homeostasis and signaling functions, including redox signaling, cell metabolism, survival, proliferation and differentiation through the modulation of transcription factors and epigenetic pathways [1]. Moreover, ROS are important regulators of inflammation and protective immune response [2]. Increased accumulation of ROS, resulting from endogenous overproduction or exogenous administration, might disrupt the naturally occurring antioxidant defense mechanisms, and lead to impaired redox homeostasis, defined as OS [3]. In this setting, excessive ROS trigger pathological redox signaling, causing cellular damage and subsequent oxidative modifications to cellular macromolecules (DNA, lipids, proteins, etc.) [4]. These deleterious oxidative effects to biomolecules cause severe damage to both structure and function (peroxidation of lipids, deactivation of enzymes, fragmentation of proteins, mutations of DNA and modifications of carbohydrates), through mechanisms that cause an accelerated aging process [5]. To counteract these deleterious effects of ROS, naturally occurring antioxidant defense mechanisms exist, which, along with exogenously administered antioxidants, work together to attack and deactivate free radicals and repair oxidative biomolecules [6]. Although the pathogenesis of OS has been known for over two decades, several issues still remain to be fully elucidated, including the underlying mechanisms, clinical effects and treatment options, in various settings [7].

In CKD and end-stage kidney disease (ESKD), research has focused on OS during the past two decades. OS occurs in early CKD stages, is gradually aggravated in parallel to the deterioration of kidney function and is an independent risk factor for cardiovascular disease (CVD), death and progression to ESKD [8,9]. Notably, oxidation of lipids might also play a regulatory role and magnify the risk of CKD progression that is conferred by proteinuria [10]. When reaching ESKD, OS is further increased and associated with adverse clinical outcomes, including loss of residual renal function, atherosclerosis, hypertension (HT), CVD and death [11]. This might be explained by the fact that cellular OS induces apoptosis, senescence and reduced regenerative capability, and triggers inflammation via the formation of pro-inflammatory oxidized lipids or advanced oxidation protein products (AOPPs), while stimulation of nuclear factor κB (NFκB) in the pro-oxidant environment promotes the expression of pro-inflammatory cytokines and recruitment of pro-inflammatory cells [7]. Moreover, the effect of OS on renal pathology lies in the accumulation of extracellular matrix proteins, podocyte damage, mesangial expansion, renal hypertrophy, endothelial dysfunction, tubulointerstitial fibrosis and glomerulosclerosis [12]. A pro-oxidant state can occur as early as in CKD stage 3, as evidenced by reported increases in circulating OS biomarkers while antioxidant systems are compromised in CKD patients and deteriorate gradually with the degree of renal function [11,13]. In ESKD, the dialysis modality plays a crucial role in the pathogenesis of OS. In hemodialysis (HD), the main culprit of OS is the bioincompatibility of dialysate, membrane and plastic tubes and connections. At the initiation of a HD session, the patient’s blood comes into contact with the synthetic, bioincompatible dialysis filter and dialysate, and the white blood cells are stimulated to produce ROS. This is why within the first 15 min, there is a 14-fold increase in ROS levels. Other factors promoting the overproduction of free radicals in HD include the use of central venous catheters, renal anemia, intravenous administration of iron and the presence of microinflammation and microorganisms in the dialysate [14,15]. Moreover, HD is also a state of impaired antioxidant defense mechanisms, due to losses of hydrophilic unbound small-molecular-weight substances, such as vitamin C, trace elements and enzyme-regulatory compounds, and dietary restrictions leading to limited dietary intake of vitamins and antioxidants [16]. On the other hand, in peritoneal dialysis (PD), the membrane used is the natural peritoneal membrane, there are no central venous catheters, iron is rarely needed and there is no blood contact; still, PD patients exhibit significantly higher OS compared to pre-dialysis CKD stage 3–4 patients, although this is lower than in HD patients. In this setting, OS is triggered mainly by the non-physiologic composition of conventional PD solutions, including their high glucose concentration, increased osmolarity and acidic pH. When the peritoneal mesothelial cells (PMCc) are exposed to the bioincompatible PD fluid, structural alterations occur, leading to peritoneal fibrosis, vasculopathy, neoangiogenesis and progressive degradation of the peritoneal membrane [17,18].

**Figure 1:**
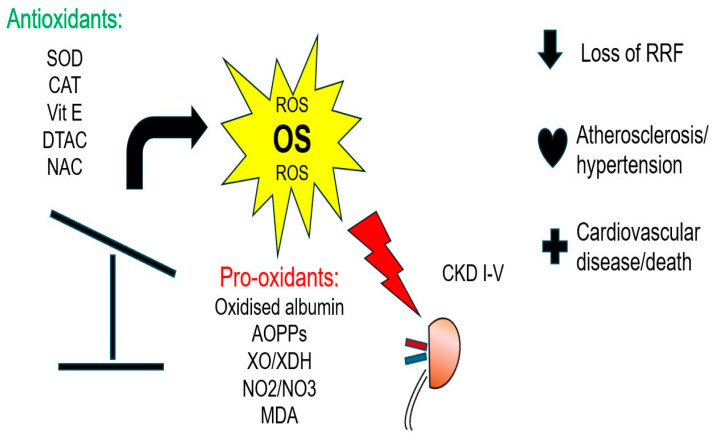
Clinical impact of oxidative stress in CKD and ESKD.

In CKD patients, the leading cause of mortality is CVD; however, this heavy CVD risk cannot be solely explained by traditional Framingham risk factors (diabetes (DM), HT, dyslipidemia) [19]. During the past two decades, accumulating evidence has suggested that OS might play a central role in the pathogenesis and progress of atherosclerosis in CKD, and should therefore be considered as a novel uremia-related risk factor for CVD [20]. The oxidative transformation of molecules such as lipids, proteins, carbohydrates and nucleic acids leads to cell apoptosis and death, triggering inflammation and the development of endothelial dysfunction, which is considered the hallmark of atherosclerosis [21]. Furthermore, OS induces the osteoblastic transformation of vascular smooth muscle cells, which is the first step towards vascular calcification (VC) [22]. Although during recent years, it has become clear that OS has a clinical impact in CKD and ESKD (Figure 1), there is still no consensus or guidelines suggesting how to measure, quantify or treat OS in these patients. This is mainly attributed to the fact that direct measurement of ROS is practically impossible, due to their low concentration, highly reactive nature and short half-life (which is usually less than a second). Thus, we measure the oxidative by-products of ROS reactions and antioxidant molecules [20]. The high heterogeneity of many OS biomarkers causes confusion and a lack of uniformity [23]. Among the most important OS biomarkers are the products of oxidative modifications of lipids, proteins and nucleic acids, as well as the measurement of the total antioxidant capacity (TAC) of the biological fluids. This review article will focus on the biomarkers of OS and the antioxidant defense mechanisms that are most clinically relevant and have been more extensively studied in patients with CKD, aiming to provide a clearer understanding of this complex issue.

## 2. Biomarkers of OS: Pro-Oxidants

**Figure 2:**
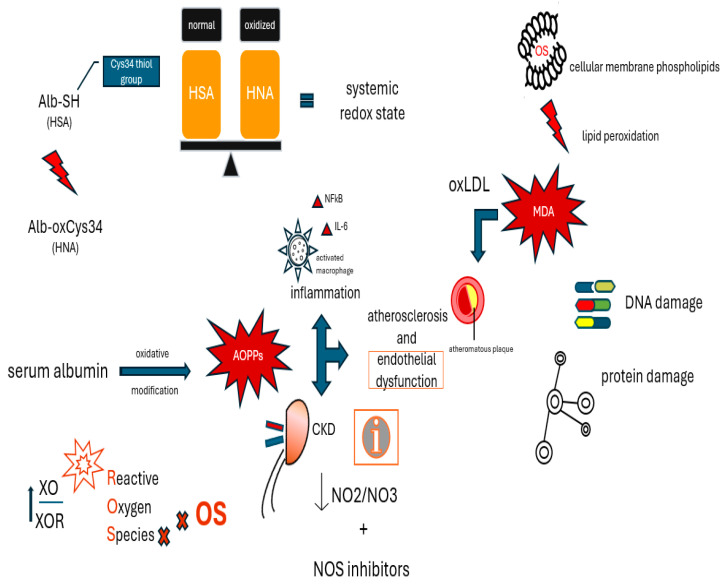
Molecular mechanisms underlying the effects of pro-oxidants.

Figure 2 shows the molecular mechanisms underlying the effects of pro-oxidants.

### 2.1. Oxidized Albumin

Human serum albumin (HSA) is a multifunctional protein that transports numerous endogenous and exogenous substances, regulates osmotic pressure and blood pH and supports antioxidative defense. The antioxidant effects of HSA result from the fact that HSA is a predominant source of free thiols (Cys34 thiol group in particular) and exhibits a high copper-binding affinity [24]. Since post-translational oxidation and glycation of HSA directly affect the Cys34 thiol group, it was hypothesized that oxidized albumin might serve as a marker of OS. Albumin containing a reduced Cys34 thiol group is called “human mercaptoalbumin” (HMA), while albumin containing a Cys34 thiol group that exists in an oxidized state is called “human non-mercaptoalbumin” (HNA) [25]. The oxidation of albumin in Cys34 has been associated with various adverse clinical effects in both the liver and kidneys [26], and levels of oxidized albumin increase in parallel with CKD progression. The ratio between the oxidized and normal forms of HSA, represented in the redox states of Cys34, serves as a marker of systemic redox state, and has been closely associated with early diagnosis and prediction of the prognosis of kidney disease [25,27]. Liu et al. [28] established a rapid and quite accurate method to quantify oxidized albumin by high-performance liquid chromatography (HPLC), and in order to validate their method, they conducted an experimental study in a rat model of albuminuria and HT. In this setting, the antioxidant Tempol was evaluated as a potential therapeutic agent to halt CKD progression, and the measurement of oxidized albumin was proposed as a practical and sufficiently validated biomarker of OS. In 5/6 nephrectomized rats with high salt intake, oxidized albumin was significantly higher in comparison to in the normal diet group, and was positively correlated with kidney function. After Tempol administration, the impact of high salt loading was reversed, leading to normal levels of oxidized albumin and inhibition of inflammatory and pro-fibrotic signaling pathways [29]. Terawaki et al. used the redox state of HSA as a marker to quantify the status of OS in 55 pre-dialysis patients, and demonstrated that OS correlates with the degree of kidney function, and is increased even in pre-dialysis CKD [30]. The HMA fraction of albumin was significantly reduced in HD patients compared to normal age-matched control subjects. Moreover, this percentage increased 3–5 h after the initiation of a single HD session, and then was reduced to subnormal levels, thus indicating that serum albumin might serve as a crucial extracellular antioxidant in HD patients, which is heavily affected by the HD procedure per se [31]. Reduced serum antioxidant activity in HD patients, assessed by an impaired HSA-redox state, contributes to the high oxidative damage seen in these patients, and is tightly associated with accelerated atherosclerotic changes and subsequent cardiovascular (CV) mortality [32]. In a prospective observational study with a long follow-up, Lim et al. showed that high levels of HNA in 249 normoalbuminemic HD patients were associated with a 2.2-fold increase in CV death risk and a hazard ratio of 1.57 for all-cause mortality, after adjusting for traditional CV risk factors [33]. Experimental data from HD patients suggest that a low albumin index due to oxidative modifications triggers an inflammatory response in endothelial cells, increasing mRNA expression of inflammatory cytokines (interleukins IL-6, IL-8 and IL-1β), and oxidized HD albumin upregulates the vascular cell adhesion molecule (VCAM) in endothelial cells [34], causing inflammation and endothelial dysfunction. Since these two entities are interrelated and both promote the development and progression of kidney disease, it might be useful to evaluate not only albumin levels, but also the albumin’s redox state, for the prognosis of kidney disease [25]. Bar-Or et al. observed a phenomenon of reduced in vitro binding of exogenous cobalt [Co(II)] to the N-terminus of HSA in ischemic conditions [35]. Ischemia-modified albumin (IMA) is a marker of ischemic OS, and has been used as an early marker in the evaluation of patients with acute coronary syndrome, but has also been evaluated in various clinical conditions, including CKD [36]. Kıyıcı et al. measured albumin and IMA levels before and after a HD session in 30 stable, maintenance HD patients, and showed that there was a significant post-dialysis increase in IMA [37]. Moreover, compared to age- and gender- healthy controls, children with CKD exhibited significantly higher IMA levels in the saliva (but not in serum or urine), and, most interestingly, the saliva concentration of IMA was correlated with standard markers of kidney function, including eGFR, stage of CKD, and serum creatinine and urea levels [38]. Circulating IMA levels were significantly increased in pediatric steroid-sensitive nephrotic syndrome (SSNS) patients compared to healthy controls, and these alterations in IMA levels were more prominent in the relapse group than in the remission group, thus suggesting that elevated IMA could indirectly reflect the degree of oxidative damage in the glomeruli [39].

Overall, oxidized albumin is a marker of OS and a kidney function index, but also a nephrotoxic molecule; therefore, the assessment of its levels might serve as a novel and exciting marker for the diagnosis and prognosis of CKD complications, including atherosclerosis, CVD and CKD progression to ESKD.

The associations of OS markers with CVD and CKD are shown in Table 1 and Table 2.

### 2.2. Advanced Oxidation Protein Products (AOPPs)

Proteins are molecular targets for oxidation reactions, because of their rapid reaction rates with oxidants and their high abundance in cells, extracellular tissues and body fluids. OS degrades lipids and carbohydrates to highly reactive intermediates, which eventually attack proteins at various functional sites. Consequently, protein oxidation, glycoxidation and lipoxidation cause a wide variety of distinct post-translational protein modifications [55]. Reversible modifications constitute signaling mechanisms under appropriate conditions (“redox signaling”), while non-reversible modifications may contribute to pathological conditions and several diseases [56]. AOPPs were identified in the plasma of uremic patients by Witko-Sarsat et al., who first described a spectrophotometric assay to quantify AOPPs in μmol/L of chloramine-T equivalents [57]. AOPPs are products generated by the attack of free radicals on serum albumin, and serve as OS biomarkers [20]. In human embryonic kidney cells, AOPPs induced the transcription of inflammatory genes (ex., NF-kB, IL-6) [58]. In streptozotocin-induced diabetic rats and in 5/6 nephrectomized rats, i.v. administration of AOPPs-RSA (AOPP-modified rat serum albumin) led to renal macrophage infiltration and overexpression of chemoattractants and pro-fibrotic agents, and led to structural and functional abnormalities, such as glomerular hypertrophy, fibronectin accumulation, accelerated fibrosis and albuminuria, probably through activation of renal NADPH (nicotinamide adenine dinucleotide phosphate hydrogen) oxidase [59,60]. The same enzymatic complex is implicated in the pathogenesis of muscle atrophy-sarcopenia. In 5/6 nephrectomized mice, AOPP overload was associated with decreased running and hanging time, a finding that was also validated in HD patients, where higher serum AOPP levels co-existed with sarcopenia [61]. Arimura et al. investigated the relationship between adipose tissue and AOPPs by conducting both in vitro and in vivo studies on adenine-induced CKD mice, and showed that AOPPs contribute to macrophage-mediated adipose inflammation, and, interestingly, the activation of inflammation could be suppressed at the gene expression level by the presence of NADPH oxidase inhibitors [62].

Declining renal function has been associated with an increase in AOPP levels, thus making them candidate markers of CKD progression [63]. Conti et al. evaluated serum levels of AOPPs in a cohort of 62 patients with DM and 56 with HT, with or without renal complications, and showed that AOPPs were increased in diabetic and hypertensive subjects compared to healthy controls, and were increased in parallel with deterioration of kidney function. The authors concluded that assessment of circulating AOPPs might predict the onset and progression of kidney disease [49]. In patients with type 1 DM, specific SOD2 allelic variations were associated with higher plasma AOPPs and progression to severe diabetic nephropathy (DN) [64]. A longitudinal study in 205 patients with CKD stages 2–5 and 40 healthy controls showed that AOPP levels increased with the decline of kidney function, and were positively correlated with the intima media thickness (IMT) of the common carotid artery. Furthermore, AOPP mean values were higher in patients with atherosclerotic plaques compared to those without plaques. The authors concluded that AOPPs might serve not only as an OS marker, but also as a surrogate biomarker for CKD progression and vascular calcification [40]. The exact mechanisms underlying these associations might not be limited only to OS, but might also include inflammation and changes in the adipose tissue. In agreement with these findings, Vinereanu et al. showed that in pre-dialysis CKD stages G3–G5, AOPPs were significantly associated with C-reactive protein (CRP), high-density lipoprotein (HDL) levels, glycated hemoglobin (HbA1c) and pulse wave velocity (PWV) values, thus suggesting that the measurement of AOPPs could be of use for CV risk assessment in pre-dialysis CKD [41]. The prognostic value of AOPP measurement has also been tested after cardiac interventions; serum AOPPs measured 1 h after cardiopulmonary bypass had a positive predictive value of 68% for predicting acute kidney injury (AKI) [65], and in a prospective study after coronary artery bypass grafting, increased AOPP levels indicated poor recovery from AKI, deteriorating overall long-term prognosis [66]. In the setting of an intensive care unit, Lentini et al. recruited 86 medical and surgical patients, and found that AOPP levels were higher in AKI compared to non-AKI critically ill patients, but their measurement could not identify those patients at risk of developing AKI within a four-day observation window [67].

The role of AOPPs in inflammation and OS in patients undergoing HD has been extensively studied. AOPPs are active inflammatory mediators through their ability to trigger oxidative burst and the synthesis of pro-inflammatory cytokines in phagocytes, and might be regarded as uremic toxins [68]. Colombo et al. reported a tight association between AOPPs and uremia in HD [69], whereas AOPPs seem to be more accurate and reliable markers of inflammation than AGEs, and correlate significantly with prothrombotic fibrinogen, thus suggesting an additional role in accelerated atherosclerosis [70]. In a cohort of 60 HD patients, AOPP concentration was significantly associated with common carotid artery IMT and wall-to-lumen ratio [42]. A large cross-sectional study on dialysis patients showed that the accumulation of AOPPs was an independent risk factor for the presence of ischemic heart disease only in the HD group, but not in the PD group [71], and an 8-year follow-up prospective study on 199 Chinese HD patients concluded that elevated serum AOPP levels were associated with a higher risk of all-cause mortality [72]. Contrary to previous findings, in another prospective study on 347 HD patients, Zuo et al. conducted a head-to-head comparison of the predictive value of four different OS biomarkers, and concluded that both AOPPs and oxidized low-density lipoprotein (oxLDL) had no impact on all-cause mortality. The authors attributed this difference to the longer follow-up period and different demographics of the aforementioned study [73].

In patients undergoing PD, AOPP levels tend to increase in parallel with the duration of the treatment. In a prospective sequential study, 22 PD patients were recruited, and serum AGEs and AOPP levels were measured prior to, and 6 and 12 months after, the initiation of the method. The rise in AGEs was attributed to the patients’ exposure to glucose-containing dialysates, whereas concerning AOPP levels, a decrease was noted [74]. Later, Gonzalez et al. measured plasma AOPP levels in 48 patients 6 months and 1 year after starting PD. The vast majority of patients showed an increase in plasma AOPP level at 1 year, and interestingly, those patients in whom the AOPP levels increased more than 50% above the baseline value had a 4.7 times greater risk of suffering a CV event compared to those with a smaller increase, even after adjusting for previous CV history [43]. In a longitudinal study, loss of residual renal function (decreased urine output and renal creatinine clearance) was associated with increased plasma AOPP levels [50]. Additionally, in a cross-sectional study of 75 PD patients, increased levels of AOPPs were independently associated with central systolic and diastolic blood pressure (cSBP, cDBP), even after adjusting for markers of fluid overload (extracellular water by multi-frequency bioimpedance, N-terminal pro-B-type natriuretic peptide-NT-proBNP), inculpating OS in the pathogenesis of HT [44]. Lastly, Furuya et al. conducted an interventional study to evaluate the effect of candesartan, an angiotensin II receptor blocker, on plasma levels of adiponectin and AOPPs in eight non-diabetic PD patients. They concluded that it decreased AOPP levels in a treatment-dependent manner, and that the increase in adiponectin levels could signify mitigation of OS and atherogenesis [75].

In CKD and ESKD, circulating AOPPs are implicated in several molecular pathways, including OS, inflammation, atherosclerosis, sarcopenia and kidney function. Therefore, the assessment of AOPPs might be applicable for the diagnosis and prognosis of CKD, AKI, CVD and mortality. In the future, large, prospective cohort studies are needed, with long follow-ups and large sample sizes, in order to fully elucidate the possible role of AOPPs in these aforementioned settings.

### 2.3. Xanthine Oxidase/Dehydrogenase

Xanthine oxidase (XO) is the oxidative radical-producing isoform of xanthine oxidoreductase (XOR), also known as urate-producing enzyme. XO is formed by reversible or irreversible oxidation of another isoform, xanthine dehydrogenase (XDH). The XO enzyme catalyzes oxidation of hypoxanthine to xanthine, and then xanthine to uric acid [76]. The XOR and XO activities both show positive correlations with eGFR; however, the ratio of XO to total XOR, known as plasma XOR redox, is inversely correlated with eGFR. A high XO/XOR ratio contributes to the elevation of OS via ROS generation, and a ratio value >1 indicates severely impaired renal function in the setting of accelerated plasma XOR conversion to XO [77].

Terawaki et al. included in their study 13 non-dialysis patients with varying degrees of kidney function, and showed that plasma XOR redox is closely related to HSA redox [78]. In a prospective study, Gondouin et al. included 51 non-diabetic patients with CKD stages 3–5 and 50 chronic HD and 38 matched healthy controls. After a 3-year follow-up time, they found that XO activity was an independent predictor of CV events in CKD and HD patients, regardless of uric acid levels, thus suggesting that the beneficial effects observed with XO inhibitors on CVD in CKD may be due to the reduction of OS [79]. Focusing on the effect of allopurinol treatment on vascular function, Sun et al. found no association between serum XO activity or endothelial XO expression and biomarkers of vascular inflammation or OS in patients with stage 3 CKD and asymptomatic hyperuricemia [80]. Additionally, in vivo studies in uninephrectomized mice and in vitro studies in human kidney proximal tubule epithelial (HK-2) cells demonstrated that XO could be a novel therapeutic target for hypercholesterolemia-associated kidney injury in uninephrectomized patients [81].

The data regarding the possible role of XO in kidney diseases are extremely limited, and are mainly derived from experimental studies. However, the idea that this OS biomarker might reflect kidney function is attractive, and warrants more research.

### 2.4. NO Synthase-Nitrite and Nitrate (NO_2_ and NO_3_)

Nitric oxide (NO) is a gaseous signaling molecule, produced by endothelial cells through the action of endothelial NO synthase (eNOS), that plays a major role in controlling vascular tone. NO production is stimulated by circulating mediators, hormonal modulators and physical factors (e.g., shear stress), while experiments in isolated blood vessels and endothelial cell cultures have shown that there is a basal release of NO, even in the absence of obvious external stimuli. When NO reaches vascular smooth muscle, it causes relaxation and vasodilation, but also inhibits platelet aggregation and white blood cells’ adhesion at the surface of the endothelial cells [82].

NO is a highly reactive molecule and has a very short half-life, so the inorganic anions formed by oxidization of NOS-derived NO-nitrate and -nitrite are measured instead. These anions are endogenously formed, but can also be found in high amounts in our daily diet. Still, they are useful indirect markers for evaluating NO production, eNOS activity and the status of endothelial function, making them important tools in clinical research related to CVD [83]. Preclinical and clinical studies have shown that reduced NO generation due to compromised eNOS (primarily found in the endothelial cells of the afferent arteriole) and/or neuronal nNOS (primarily expressed in the macula densa cells) activity can increase preglomerular resistance, reduce glomerular perfusion and filtration and increase tubular reabsorption of sodium, which subsequently lead to the development or progression of HT and kidney damage [84,85]. Using a rodent model, Prabhakar et al. found that in DN, both renal NO production and expression of renal NOS were decreased, while markers of mitochondrial OS (8-hydroxydeoxyguanosine) were upregulated. Interestingly, the administration of the antioxidant alpha-lipoic acid could reduce proteinuria and OS, and treatment of hyperglycemia could delay the progression of CKD [86]. Reduced eNOS immunoreactivity and endothelial dysfunction preceded capillary loss and led to worsening of renal disease in experimental polycystic kidney disease [87]. The enhancement of NO bioavailability in CKD rats has been tested with the use of antioxidant mixtures (L-carnitine, Catechin, vitamins E and C) [88] and sodium hydrosulfide [89], providing evidence that certain interventions could exert renoprotective effects by attenuating inflammation and OS, and could restore kidney function.

In a large cohort of 375 participants, Kleinbongard et al. showed that plasma nitrite levels were tightly correlated with surrogate markers of endothelial dysfunction (flow mediated dilation (FMD), carotid IMT) and with traditional, Framingham CV risk factors (HT, dyslipidemia, smoking, advanced age). Furthermore, there was a progressive reduction in plasma nitrate with an increasing score of the aforementioned CV risk factors [45]. In patients with isolated essential HT, the presence of constitutive NOS (cNOS) inhibitors and lipid peroxidation products was found to worsen blood pressure control and lead to microvascular endothelial dysfunction [90]. Therapeutic strategies that correct the imbalance between the production of ROS and NO may have clinical implications. Oral nitrate ingestion was found to have a consistent BP-lowering effect in both healthy volunteers and hypertensive patients [91], possibly through antioxidative effects via different targets and cellular mechanisms (modulation of mitochondrial function, reduction in NOX- and XO-derived ROS production, restoration of eNOS function). Through the improvement of endothelial dysfunction and OS, nitrate treatment might delay the progression of cardiorenal complications [92].

In CKD, inflammation, OS, mineral bone disease (low vitamin D, hyperphosphatemia, high fibroblast growth factor 23 (FGF23) and low Klotho) and the accumulation of NOS inhibitors all contribute to reduced NO bioavailability and endothelial dysfunction [21,93], resulting in the development and progression of CVD [92]. In a small observational study on 27 ESKD patients, reduced NO bioavailability post-dialysis, confirmed by measuring plasma levels of nitrates and nitrites, was associated with increased CV mortality [94]. In PD patients, NO levels were decreased compared to healthy controls, irrespective of peritoneal transport status, but there was an interesting negative correlation between NO and the peritoneal equilibration test (PET) that could suggest alterations in the bioavailability of NO in the vascular endothelium in this setting [95].

In living kidney donors, NO biomarker improvement begins on the first day of kidney transplantation, and NO levels on day six could be a predictive marker of eGFR at six months [51]. Nitrite therapy might be beneficial during the procedure of kidney transplantation, as suggested by experimental evidence that topically administered sodium nitrite protects the kidney from ischemia/reperfusion injury via the generation of XOR-catalyzed NO [96].

NO plays a pivotal role within the endothelium, and therefore reflects endothelial dysfunction, the hallmark of atherosclerosis. The clinical plausibility of this OS marker relies on the fact that besides diagnosis and prognosis, NO might also serve as a therapeutic target in kidney and CVD.

### 2.5. Malondialdehyde (MDA)

MDA is a highly reactive and toxic aldehyde, generated by polyunsaturated fatty acid peroxidation or prostaglandin breakdown by cyclooxygenase activity, or via various non-lipid precursors, involving amino acids and carbohydrates [97]. MDA is an established marker of lipid peroxidation status, thus indicating OS and early atherogenesis in both clinical research and epidemiological studies [98]. On a molecular level, lipid oxidation of cell membranes releases MDA and causes impaired elasticity and limited membrane function. MDA forms covalent bonds with biomolecules, especially proteins and nucleic acids. The assay to determine MDA in tissues or blood involves the reaction of MDA with Thiobarbituric acid to form TBARSs (thiobarbituric acid reactive substances) in an acidic medium [99]. MDA, like other reactive aldehyde species (4-hydroxy-2-nonenal (HNE), acrolein), is generated within enzymatic pathways that lead to post-transcriptional alterations in DNA and proteins, eventually resulting in genotoxicity, inhibition of gene expression, cytotoxicity and cellular death [97]. In vitro and in vivo studies have coherently showed that MDA is toxic to cells and tissues [100], promotes atherogenesis [101] and is heavily implicated in the pathogenesis of chronic inflammation [102].

In human studies, higher serum MDA levels were found in CKD patients compared to healthy controls, while MDA was negatively correlated with eGFR. Tomás-Simó et al. conducted a prospective study in 155 non-dialysis patients at different stages of CKD and 45 healthy controls, and found a progressive increase in circulating levels between controls and CKD stage 1, and among CKD stages (2 versus 1, 3 versus 2, 4 versus 3) [52]. In agreement with these findings, in 402 patients with DN, serum MDA-modified LDL (MDA-LDL) levels were significantly increased in severe albuminuria, and an increased MDA-LDL/LDL ratio was significantly associated with both increased albuminuria and reduced eGFR, thus suggesting that this marker is implicated in the progression of DN [53]. Moreover, among 120 CKD pre-dialysis patients, the presence of DM type 2 was associated with significantly increased plasma MDA levels [103]. Table 3 shows the differential expression of MDA and other OS markers in CKD/ESKD patients and healthy controls.

In HD patients, MDA levels are higher than in healthy controls matched for age, gender and body mass index (BMI) and pre-dialysis CKD patients [106,107]. This might not be solely attributed to disease progression; it has been shown that MDA levels do not differ significantly between CKD stages 4 and 5 [52]. Therefore, the effect of a single HD session on MDA levels has been evaluated in several small single-center studies. MDA is a water-soluble compound with a low molecular weight, and thus is expected to diffuse across HD membranes. On the other hand, each HD session triggers acute autoimmune activation reactions and causes abrupt generation of free radicals, which, in turn, might interact with cell membrane lipids to produce MDA. After accounting for the clearance of MDA through the membrane, Lakshmi et al. found a significant increase in circulating MDA from pre to post in a single HD session, thus indicating that HD modality, overall, triggers lipid peroxidation [108]. In agreement with this finding, a study that included 20 patients on HD, 16 patients on CAPD and 20 healthy controls showed that MDA was elevated in post-HD and CAPD patients in comparison to pre-HD and control groups, and the increased lipid peroxidation was accompanied by low levels of the antioxidant glutathione [104]. However, a small observational study that investigated the effect of plasma vitamin concentrations on markers of OS during HD showed that the plasma MDA concentration did not change significantly, while vitamin C levels decreased by 40% after a single HD session [109]. Steghens et al. provided a methodological explanation by measuring true total and free plasma MDA in HD patients, before and after the sessions, by the HPLC method, concluding that free MDA is bound to low-molecular-weight compounds, and total MDA is mainly produced during HD sessions [110].

Besides kidney function and OS, MDA might serve as a surrogate marker of endothelial dysfunction and sarcopenia. In HD, MDA was inversely associated with performance on 6 min walk test (a measure of functional capacity) [111], and was positively associated with carotid IMT, left ventricular hypertrophy [112], PWV and vascular stiffness [46,47,105]. Specifically, serum MDA-modified LDL, along with DM and HT, were independent predictors of arterial stiffness (AS) (defined by carotid-femoral cfPWV) in HD patients [46], whereas ROC (receiver operating characteristic) curve analysis showed that the critical cut-off value of 80.91 mg/dL for MDA-LDL predicted peripheral arterial stiffness (PAS) with accuracy [47]. The authors concluded that not only does MDA plays a role in the pathogenesis of arterial stiffness, but it could also be considered as a therapeutic target in this condition. Interestingly, the presence of antibodies to MDA-LDL had no correlation with thromboembolic events [113]. In 39 HD patients, pre-dialysis serum MDA concentrations were correlated with coronary artery calcification (CAC—calculated by multirow spiral computed tomography), and those patients with the highest MDA levels were four times more likely to have severe CAC [48]. In a cross-sectional study, Boaz et al. concluded that serum MDA levels, both before and after the midweek session, were the strongest predictor of CVD prevalence, while other known risk factors, such as serum lipids and plasma fibrinogen, failed to show any association [114,115].

Patients on PD have higher MDA levels compared to pre-dialysis CKD patients, but lower MDA levels compared to HD, which can be attributed to the fact that PD is a more biocompatible dialysis modality than HD [116]. In contrast to HD, the presence of DM in PD patients did not significantly affect MDA levels [117]. However, PD efficiency, assessed by total weekly Kt/V, has been negatively associated with circulating MDA [118]. The culprit for this might be the conventional peritoneal dialysates that have low pH, high osmolarity, and high glucose, GDPs and AGEs. Chronic exposure of the peritoneal membrane to glucose gradually leads to neo-angiogenesis, thickening of the membrane, inflammation and OS and apoptosis of the mesothelial cells. This hypothesis is also supported by the fact that 6 months of use of icodextrin-PD solutions (which is a non-glucose, osmotic colloid agent) caused a significant decrease in MDA levels [119].

Kidney transplantation partially restores kidney function, and this seems to cause a significant and time-dependent decrease in MDA levels, which leads to normalization within the first 3 years post-transplantation [120]. A prospective study in 40 kidney transplant recipients showed that increased MDA levels on the first day postoperatively might be an early prognostic indicator of delayed graft function (DGF), and if increased MDA persists after 7 days, this might be a negative predictor of 1-year graft function [54].

Various pharmacological and non-pharmacological approaches, including antioxidant supplementation, lifestyle modifications and drug therapies, have shown potential in mitigating oxidative damage by reducing MDA levels in patients with kidney disease. In a meta-analysis of seven randomized controlled trials (RCTs), including 392 HD patients, probiotic, prebiotic and synbiotic supplementation effectively reduced the levels of circulating uremic toxins and inflammatory biomarkers, while restoring the balance between antioxidant and pro-oxidant markers, with a significant reduction in MDA levels in the intervention group when compared with the placebo group [121]. Also, aerobic exercise managed to successfully suppress circulating MDA in CKD patients [122], thus highlighting the beneficial antioxidant effects of chronic exercise in these patients.

MDA plays a central role in the lipid peroxidation of biological membranes. However, multiple other roles in the pathogenesis and progression of various conditions have been proposed for this oxidant, including CVD, arterial stiffness, CKD, inflammation and sarcopenia. Besides serving as a marker of diagnosis and prognosis, MDA might also serve as a therapeutic target in dialysis populations.

## 3. Biomarkers of OS: Antioxidants

**Figure 3:**
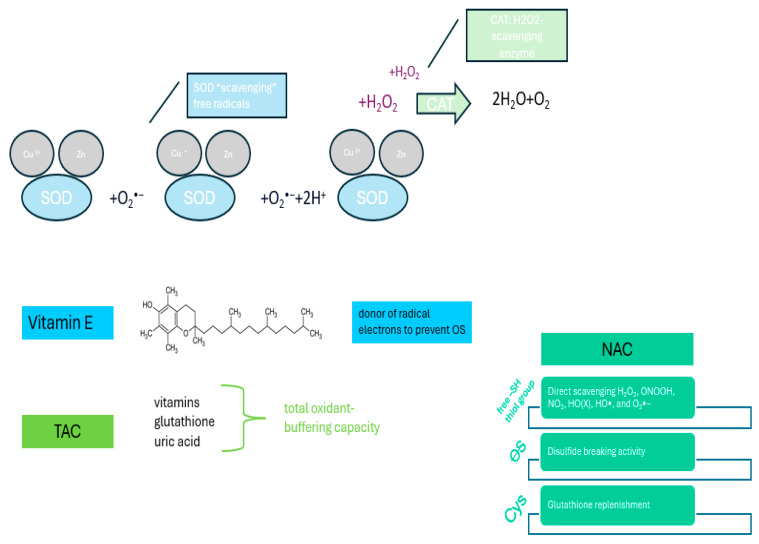
Molecular mechanisms underlying the effects of antioxidants.

To counteract the deleterious effects of free radicals and pro-oxidants on biomolecules, there are both naturally occurring and endogenously and exogenously administered antioxidants. The main mechanisms underlying the effects of antioxidants are shown in Figure 3.

### 3.1. Superoxide Dismutase (SOD)

There are three mammalian SODs: copper–zinc SOD (SOD1) and manganese SOD (SOD2), which exist exclusively in the intracellular environment, and extracellular SOD (EC-SOD), primarily localized in the extracellular space. EC-SOD is an antioxidant enzyme expressed in abundance in the kidney, and has a special role in the homeostasis of the kidney and the vasculature, mainly by regulating the free radical superoxide anion, which is generated in renal disease through upregulation of NADPH oxidases or a leak from the mitochondrial electron transport chain [123]. All SODs catalyze the dismutation reaction of the superoxide anion free radical to hydrogen peroxide and normal oxygen [124]. Therefore, the strong antioxidant effects of SOD lie in its ability to directly scavenge free radicals. SOD has been associated with local kidney oxidation in CKD, whereas in dialysis, the uremic environment is hypothesized to significantly downregulate both the function and concentration of SOD.

The first reports regarding the association of SOD with kidney function date from three decades ago. Kashem et al. suggested that low levels of SOD activity (which might be either the cause or the result of glomerulonephritis) lead to reduced local antioxidant defense, oxidation of the kidney cells and deterioration of kidney function [125]. Since then, the significance of SOD has been studied in various settings. SOD2 dysfunction has been reported to aggravate renal damage and apoptosis in the kidney at the cellular level due to mitochondrial OS [126].

In animal models, EC-SOD knockout mice showed attenuated renal blood flow and decreased SOD activity after kidney ischemia/reperfusion [127], while Rodríguez-Iturbe et al. showed that SOD2 deficiency was associated with renal interstitial inflammation and accelerated glomerulosclerosis, tubulointerstitial damage and salt-sensitive HT, especially in aged mice [128]. SOD has also been associated with albuminuria; downregulation of renal SOD1 and SOD3 was observed in progressive DN [129], and finerenone, a novel non-steroidal mineralocorticoid receptor antagonist, achieved an increase in renal SOD activity and a reduction in albuminuria in CKD animal models [130].

In a retrospective study of 134 hypertensive patients with microalbuminuria, Yu et al. found a negative correlation between SOD and urine albumin–creatinine ratio (UACR), and thus, SOD was proposed as an independent protective factor against early renal damage in this group of patients [131]. There is a J-shaped pattern of circulating SOD across CKD stages: SOD is progressively reduced in stages 1–4, and then increases in ESKD [132]. Moreover, the expression of the SOD2 gene was similar between groups, whereas increased SOD2 protein content was associated with an increased risk of all-cause mortality in the HD group. The gradual decline in SOD2 protein content across CKD stages 1–4 might be explained by the fact that OS, inflammation and uremia all contribute to excessive protein degradation. OS and inflammation act synergically to release free radicals in CKD, which, in turn, downregulate SOD2 protein content. Additionally, through activation of the ubiquitin-proteasomal pathway, the uremic environment triggers further degradation of SOD2 protein content. The exact mechanisms underlying the abrupt increase in SOD2 in ESKD have not yet been fully elucidated. In uremic animals, supplementation of vitamin D receptor antagonists (VDRAs) successfully reversed SOD2 protein upregulation. Therefore, the authors hypothesized that since ESKD is characterized by poor vitamin D status, the use of VDRAs might partially explain the J-shaped pattern of SOD2 protein content. When compared with controls, HD patients present decreased SOD activity, which is probably influenced by zinc nutritional status [133], and this reduced antioxidant activity is negatively correlated with plasma MDA levels [134]. In a survival analysis, HD patients with higher SOD activity had a survival benefit, suggesting that SOD can serve as a predictor of all-cause and CV mortality [135]. A genotype–phenotype association study involving 178 HD patients showed that a specific EC-SOD polymorphism (EC-SOD Arg213Gly) could accelerate OS and atherosclerosis in its carriers, demonstrated by higher IMT and plasma OxLDL values [136].

Studies on PD patients showed increased SOD activity, irrespective of the peritoneal membrane transport status, when compared with healthy controls, possibly in an attempt to balance OS, while the levels of the DNA repair enzyme hOGG1 (human 8-oxoguanine DNA glycosylase 1) and TAC were both significantly decreased [95]. When measuring SOD activity in the erythrocytes of ESKD patients, a significant decrease was observed in HD patients using polyacrylonitrile or cuprophane membranes, whereas enzymatic activity increased in patients undergoing CAPD [137]. Inhibition of SOD activity in the red blood cells (RBCs) of dialysis patients might also contribute to renal anemia [138]. Therapeutic approaches that involve enhancing SOD activity are under investigation. SOD-gliadin supplementation in 28 patients undergoing HD was found to significantly decrease serum tumor necrosis factor α (TNF-α) and tumor growth factor β (TGF-β) levels [139]. The marked increase in Cu/Zn-SOD production in HD patients, as a response to OS and inflammation, suggest that SOD might also serve as a potential therapeutic target. In a small interventional study, Washio et al. studied the effect of orally administered vitamin C on Cu/Zn-SOD levels in 16 HD patients, but this vitamin supplementation did not effectively suppress expression enhancement [140]. In disagreement with these, vitamin E supplementation and the use of vitamin E-coated membranes (VECMs) for 6 months both resulted in a progressive decrease in Cu/Zn-SOD mRNA in the leukocytes of HD patients to the level of non-dialyzed CKD patients [141].

### 3.2. Catalase (CAT)

Among ROS, hydrogen peroxide (H_2_O_2_) is an effective secondary messenger that can diffuse to different cell compartments and the extracellular space, and catalase (CAT) is probably the most effective H_2_O_2_-scavenging enzyme [142]. Polymorphisms in genes encoding CAT have been associated with a decreased risk of CKD [143], while in vitro studies suggest a protective effect of CAT on VC induced by calcium and phosphorous [144]. Experimental CKD (5/6 nephrectomy) is accompanied by a reduction in CAT activity [145], which promotes the accumulation of collagen, fibrosis, inflammation, reduced kidney function and the onset of albuminuria [146].

Clinical data also suggest a close association of CAT with kidney function [147] and CVD [148]. In HD patients, the bioincompatibility of the modality aggravates OS by generating pro-oxidants and reducing antioxidants. Small interventional studies have focused on the effect of dialysis membranes on erythrocyte CAT activity. When HD patients were dialyzed with the newest, more biocompatible polysulfone membrane, a significant increase in CAT levels was observed [149]. CAT activity was higher in all ESKD patients (undergoing HD or CAPD) when compared to healthy controls, and an enhancement in CAT activity was observed over the course of several months in renal replacement therapy, indicating the significant exposure of patients’ erythrocytes to OS [137]. In PD patients, Stępniewska et al. investigated platelet antioxidant activity, and found that CAT activity was significantly higher in PD compared to HD patients [150]; however data on PD patients are extremely limited.

In recent years, nanobiotechnology has attempted to create nanozymes (nanomaterials exhibiting natural enzyme-like activity) in order to effectively supplement antioxidants. In an experimental study, Choi et al. developed an inflammation-sensing, ROS-scavenging versatile nanoplatform by stably loading CAT-mimicking dMn3O4 nanoparticles inside ROS-sensitive nanomicelles (PTC), resulting in an ROS-sensitive nanozyme. After i.v. administration of the nanozyme to mice with ischemia/reperfusion AKI, they observed an attenuation of inflammation and apoptosis in kidney cells [151], thus suggesting that high-performance ROS depletion strategies are yet to be developed.

### 3.3. Vitamin E

Vitamin E (or alpha-tocopherol) is a fat-soluble vitamin known for its antioxidant properties. It plays a role in the detoxification chain of fat-soluble to water-soluble toxic radicals, thus protecting various tissues from lipid peroxidation. Through its protein-dependent functions and gene modulation effects, it inhibits the production of pro-inflammatory cytokines, activates protective pathways like Nrf2 and modulates the activity of immune cells (ex., T-cells, macrophages) [152]. Specifically, vitamin E blocks monocyte O_2_ discharge, IL-1β and TNF-α synthesis, neutrophil chemotaxis and their adhesion to the endothelium, which all contribute to atherogenesis [153], and it preserves the integrity of the glomerular basement membrane [154].

The renoprotective effect of vitamin E supplementation was studied in a double-blind, randomized cross-over trial, where 30 type 2 diabetic patients with microalbuminuria were treated with combined oral supplementation of vitamin C and E. UACR was reduced by 19% after 4 weeks, even though the participants had stopped treatment with ACE inhibitors before randomization [155]. However, in a large observational study on 127,081 Korean adults with preserved renal function, dietary intake of vitamins C, E and A was not significantly associated with the risk of CKD progression [156].

CKD is a state of deficiency and impaired metabolism of vitamin E. Defects in the intake, metabolism and antioxidant function of vitamin E contribute to the premature aging of CKD patients, increasing the uremic tissues’ susceptibility to OS (a phenomenon called “inflammaging”) and abnormal lipid peroxidation [157,158]. The potential benefits of vitamin E supplementation in CKD patients have been studied in various clinical trials, especially after Rasool et al. found an association between low serum vitamin E levels and CVD in ESKD patients [148]. In a post hoc analysis of the HOPE study (Heart Outcomes Prevention Evaluation), the 993 participants with mild–moderate CKD at high CV risk saw no apparent benefit on CV outcomes with daily vitamin E supplementation [159], and a large meta-analysis of 19 clinical trials with different study populations showed that high-dosage (≥400 IU/day) vitamin E supplementation may increase all-cause mortality, and therefore, its supplementation in chronic conditions should be considered with caution [160].

The SPACE study showed that high-dose vitamin E supplementation (800 IU/day vitamin E) in 196 HD patients with pre-existing CVD significantly reduced the risk of CVD events (especially myocardial infarction) compared to a placebo group [161]. Based on these findings, and to counteract the oxidative burst that is triggered from the blood coming into contact with artificial plastic materials during HD sessions, VECMs were developed. These membranes consist of a multilayer membrane bearing a hydrophilic polymer that strongly binds liposoluble vitamin E on the blood surface. In this way, the polymer acts as a powerful radical scavenger, protecting plasma lipids and cell membranes from peroxidative events. Furthermore, VECMs are more biocompatible and exert an in situ effect on blood cells, leading to decreased activation of circulating leukocytes, decreased endothelial cell activation and decreased OS [162].

Compared to conventional dialyzers, HD with VECMs significantly decreased inflammation (CRP), OS (oxLDL) and endothelial activation (soluble intercellular adhesion molecule, sICAM-1) [163], whereas a meta-analysis of 60 RCTs showed that VECMs improved the Erythropoetin (EPO) Resistance Index and reduced inflammation and OS, as assessed by a significant decrease in IL-6 levels, TBARS, plasma and RBC MDA, while providing similar dialysis adequacy, in comparison with conventional HD membranes [164]. However, the use of VECMs failed to show any beneficial effects on lipid profile parameters or carotid IMT in HD patients [165]. More recently, a novel idea of developing nanoparticles for targeted delivery of vitamin E to renal injury cells after ischemia/reperfusion [166] has emerged, suggesting that the role of vitamin E in kidney disease remains scientifically relevant and holds potential for future clinical applications.

Vitamin E is one of the most powerful antioxidants and a promising therapeutic target in HD. However, it should be noted that the results regarding the potential beneficial effects of its supplementation in HD are contradictory, and high-dosage (≥400 IU/day) vitamin E supplementation may increase all-cause mortality. The exact mechanisms underlying this association are not yet known, but its supplementation in chronic conditions should be considered with caution. This is why, currently, the KDIGO guidelines do not recommend vitamin E supplementation in ESKD patients. The use of VECMs is attractive because they significantly improve the biocompatibility of HD and might be clinically beneficial for HD patients. However, we need future RCTs with hard clinical endpoints (such as mortality and CVD) to evaluate the potential benefits and safety of this therapeutic approach.

### 3.4. Total Antioxidant Capacity (TAC)

TAC, also known as non-enzymatic total antioxidant capacity (vitamins, glutathione and uric acid), encompasses the synergistic interaction effects of all antioxidants in a given matrix (diet/foods or body fluids), and its measurement remains one of the most widely used methods to quantify the possible oxidant-buffering capacity of an organism [167]. The overall antioxidant capacity of the diet, which can be computed by a new tool called the dietary TAC (DTAC), defines the synergistic actions of all antioxidants, and has been associated with various clinical outcomes [168,169].

Abbasi et al. studied 210 (102 cases and 108 controls) patients with type 2 DM, classified based on their CKD status, and estimated DTAC based on the ferric-reducing antioxidant power of selected foods. No significant association was found between DTAC and CKD, thus suggesting that the effects of DTAC on the risk of CKD require further validation [170]. However, a larger study on 1747 subjects with CKD found that participants with higher DTAC scores had a higher GFR, while no association was revealed between higher DTAC and kidney stone formation [169]. Moreover, diets high in TAC were associated with a lower risk of incident CKD among subjects with hyperglycemia after 3 years of follow-up [171], but this association might be weakened in the elderly, suggesting that certain molecular mechanisms, such as OS, are heavily degraded with advanced age [172].

DTAC was associated with lower all-cause mortality among patients with CKD stages 1–2, but there was no apparent benefit for patients with stages 3–5 CKD or albuminuria, implying that as uremia progresses, more contraindicatory mechanisms might be activated [173]. Every HD session is accompanied by a significant decrease in TAC [174], which can be significantly increased via engagement in chronic intradialytic CV exercise [175].

### 3.5. N-Acetylcysteine (NAC)

NAC is one of the most widely studied antioxidants in clinical studies, animal experiments and cell culture experiments. NAC is a thiol-containing agent that acts as an antioxidant through various mechanisms; it scavenges oxidants directly, acts as a source of cysteine (Cys) for increased glutathione (GSH) biosynthesis and increases sulfane sulfur production [176]. NAC has been widely used as a mycolytic and for the prevention of contrast-induced nephropathy (CIN) [177]. Moreover, NAC is the mainstay of treatment for paracetamol overdose/poisoning. By restoring hepatic glutathione stores, NAC is a safe and quite effective antidote for paracetamol-induced hepatotoxicity. The antioxidant and anti-inflammatory effects of NAC have been studied mainly in experimental models. Medipally et al. found that NAC treatment could mitigate the degree of interstitial fibrosis/tubular atrophy in 5/6 nephrectomized mice with chronic hematuria by reducing OS in the kidney [178]. In an experimental DM model, NAC exhibited protective effects against DN [179], but in an attempt to associate OS parameters with mineral bone disorder in a rat model of progressive CKD, NAC failed to significantly improve bone architecture/geometry/mechanical properties [180].

Although the clinical data regarding the effect of NAC administration in CKD patients are limited, NAC has been studied as a renoprotective agent. Chiu et al., in a retrospective study, divided 554 patients with CKD stages 3–5, 1:1, into 2 groups: NAC users/NAC non-users. After 3 years, they reported that the incidence rate of reaching HD was significantly lower in NAC users than in non-NAC users, due to the modulation of serum creatinine and eGFR levels [181]. In a small placebo-controlled study with 20 non-diabetic patients with proteinuria and mildly decreased kidney function (up to stage III), oral supplementation of NAC—as an add-on therapy to renin–angiotensin–aldosterone system (RAAS) blockade—had no effect on proteinuria, surrogate markers of tubular injury or renal fibrosis [182]. A meta-analysis by Ye et al. showed that NAC supplementation is safe for CKD patients, and there was evidence of benefits on kidney function, inflammation markers and CV risk reduction [183].

In HD patients, oral NAC managed to reduce HSA-AOPP, the subsequent phagocyte oxidative responses [184], serum MDA [185] and plasma ADMA levels [186]. A systematic review by Coombes et al. concluded that NAC was the most efficacious supplement for ameliorating OS in patients undergoing HD [187]. Apart from its antioxidant properties, NAC has shown a favorable effect on uremic anemia. With a stable EPO dosage, NAC users on stable HD had a significant increase in hematocrit, accompanied by a decrease in plasma levels of 8-isoprostane and oxLDL, after 3 months of thrice-daily supplementation [188]. Finally, a prospective, randomized, placebo-controlled trial that included 134 maintenance HD patients showed that those in the NAC group (oral administration of NAC 600 mg/day) had a 40% reduction in the risk of reaching the primary CV endpoint (a composite variable consisting of cardiac events including fatal and nonfatal myocardial infarction, CVD death, need for coronary angioplasty or coronary bypass surgery, ischemic stroke, peripheral vascular disease with amputation or need for angioplasty), for a median follow-up period of 14.5 months [189].

In the setting of PD, experimental studies have provided some exciting results. The addition of NAC into the high-glucose compartment of neutral-pH-type PD fluids has been associated with a reduction in glucose degradation products, the main culprit of peritoneal membrane failure [190]. When human PMCs are exposed to high-dialysate glucose, there is a reduction in intracellular glutathione concentration, leading to cell dysfunction and apoptosis through mitochondrial damage, a process that can be avoided if NAC is added to the cell culture [191,192]. In patients undergoing PD, oral NAC supplementation was associated with a decrease in inflammatory biomarkers [193,194] and a positive effect on residual renal function [195]. However, in a placebo-controlled study that included 30 patients on regular PD, NAC treatment did not significantly affect OS markers (AOPPs, ADMA, etc.) [196], calling into question the antioxidant benefit of NAC use in PD patients.

Among all the available methods, NAC is probably the most promising exogenously administered antioxidant in HD and PD. Through its antioxidant, anti-inflammatory and anti-atherosclerotic effects, NAC has been associated with improved outcomes in these populations and no serious side effects. However, the existing data are of low quality, derived from either experimental or clinical studies with small sample sizes, short follow-up periods, surrogate endpoints and large heterogeneity in design. Currently, there is no consensus or guidelines recommending the supplementation of NAC in ESKD. However, this is an area of research of scientific interest. In the future, large RCTs examining clinical hard endpoints are expected to shed some light on this area.

## 4. Conclusions

Although OS has been at the center of research efforts for more than two decades, there are still more questions than answers in the case of CKD and ESKD. Free radicals and ROS are highly reactive and have a half-life of milliseconds, making them practically impossible to quantify. Alternatively, pro- and antioxidants are measured as surrogate markers of OS. However, there is no consensus on which markers to measure and how often in CKD and dialysis patients. Moreover, there is no universally accepted standard for measuring OS. Different laboratories may use different techniques, reagents or protocols to assess the same marker, leading to inconsistencies in results. Additionally, OS markers can vary widely between individuals, due to factors such as age, diet, lifestyle and underlying health conditions, which can further complicate comparisons across studies. The main drawback is that the reported correlations of various OS markers with diseases and conditions such as CVD and CKD are derived from small, low-quality observational studies, and thus, no causality can be established. Moreover, it is not clear whether OS is the cause or the consequence of CKD progression. Several conditions that predispose individuals to CKD onset and progression, such as advanced age, hypertension, obesity and diabetes, also trigger OS. On the other hand, OS, along with inflammation, has been shown to promote endothelial dysfunction, vascular calcification and CKD. Therefore, it could be hypothesized that the association between CKD and OS might be a chicken and egg paradigm.

Another unresolved issue is the fact that the clinical importance and relevance of OS in CKD settings have not yet been fully elucidated. In the future, larger RCTs examining the effect of exogenously administered antioxidants on hard clinical endpoints might shed some light on this area. In order to draw definitive conclusions for the possible clinical utility of OS markers, these trials should have a large sample size, long follow-up and homogeneity regarding the populations and the markers that are examined. Another issue that remains to be examined in future studies is the safety and adverse effects of antioxidant supplements in both CKD and ESKD populations. Finally, since in HD and PD, the main culprits of increased OS are the biocompatibility of the modalities, we desperately need the development of new, more biocompatible PD fluids and HD membranes, which will be examined in large, multicenter prospective studies. The data from these future studies will help us to understand the pathophysiology of OS, the clinical impact on hard clinical endpoints and the potential benefit from antioxidant supplementation in uremic patients.

## Figures and Tables

**Table 1:** Associations of OS markers with CVD indices in CKD and ESKD.

Indices of CVD	Molecule	Study Type	Study Population	Outcome	Reference
CVD death	Fraction of HMA	Observational prospective	86 HD patients	Patients with pre-HD f(HMA) < 40%: adj. OR 2.5, patients with post-HD f(HMA) < 60%: adj. OR 25.6	Terawaki et al. [32]
CVD death	HNA fraction of HSA	Observational prospective	249 HD patients	HNA level > 51.16% is associated with 2.2-fold increase in CVD death risk	Lim et al. [33]
IMT, atherosclerotic plaques	Plasma AOPPs	Observational prospective	205 CKD patients/40 controls	Mean AOPP increased with IM diameter (<0.05) and was higher in patients with plaques (*p* < 0.05)	Azouaou Toualbi et al. [40]
PWV	Plasma AOPPs	Cross-sectional	46 pre-dialysis patients (stages G3–G5)	PWV and AOPP were positively correlated after adjusting for SCr (*p* = 0.01) and eGFR (*p* = 0.02)	Vinereanu et al. [41]
IMT	Plasma AOPPs	Observational	79 HD patients	IMT increased significantly with AOPP levels (r = 0.07, *p* = 0.02)	Drüeke et al. [42]
CVD risk	Plasma AOPPs	Observational prospective	48 PD patients	>50% AOPPs levels increased above baseline: 4.7× risk of later CVD	Gonzalez et al. [43]
cSBP, cDBP, pSBP, pDBP	Plasma AOPPs	Cross-sectional	75 PD patients	AOPPs correlated positively with cSBP (*p* < 0.05), cDBP (*p* < 0.001), pSBP (*p* < 0.01) and pDBP (*p* < 0.001)	Xu et al. [44]
IMT, FMD	Plasma nitrite	Cross-sectional	351 regular blood donors/20 healthy individuals	Plasma nitrite levels were positively correlated with FMD (*p* = 0.001) and inversely correlated with IMT (*p* < 0.01)	Kleinbongard et al. [45]
AS, cfPWV	Serum MDA-LDL	Observational	155 HD patients: 68 AS sufferers, 87 controls	MDA-LDL was independent risk factor for developing AS (OR: 1.014, *p* < 0.001)	Hou et al. [46]
PAS, baPWV	Serum MDA-LDL	Cross-sectional	100 HD patients: PAS group: 52, control group: 48	Higher serum MDA-LDL levels were independently associated with PAS in HD patients (OR = 1.014, *p* = 0.009)	Liu et al. [47]
CAC	Pre-HD MDA	Observational	39 HD patients	Patients in highest tertile of MDA were 4× more likely to have severe CAC	Jung et al. [48]

**Table 2:** Associations of OS markers with CKD indices in CKD and ESKD.

Indices of Kidney Function	Molecule	Study Type	Study Population	Outcome	Reference
eGFR	Saliva IMA	Observational	24 CKD children/24 healthy children	In CKD children, IMA was negatively correlated with eGFR (*p* ≤ 0.0001)	Szulimowska et al. [38]
Proteinuria in SSNS	Serum IMA	Cross-sectional	70 children with SSNS/45 healthy controls	IMA was significantly higher in SSNS-relapse group vs. SSNS-remission and control groups (*p* < 0.05)	Cakirca et al. [39]
UACR	Plasma AOPPs	Observational	62 type 2 diabetic patients/30 healthy controls	AOPPs levels were significantly higher in DM microalbuminuric patients (*p* < 0.05 vs. DM, *p* < 0.0001 vs. controls)	Conti et al. [49]
Serum creatinine	Plasma AOPPs	Observational	56 hypertensive patients/30 healthy controls	AOPPs levels were significantly higher in HT patients (*p* < 0.01) and CKD secondary to HT patients (*p* < 0.0001) vs. controls	Conti et al. [49]
Creatinine clearance	Plasma AOPPs	Observational, prospective	31 PD patients	Renal CrCl was inversely correlated with AOPP (*p* < 0.05)	Furuya et al. [50]
eGFR/graft function	NO	Prospective	32 transplant recipients	NO at day six predicted graft function at six months and eGFR at day 6, day 21 and 3 months post-Tx	Izemrane et al. [51]
eGFR	MDA in MN cells	Cross-sectional	155 CKD patients (stages 3–5)/45 healthy controls	MDA differences were statistically significant (*p* < 0.001) between all groups, except between CKD 4 and 5 (*p* = 1000)	Tomás-Simó et al. [52]
Albuminuria	Serum MDA-LDL	Retroactive cross-sectional	402 type 2 diabetic patients	MDA-LDL levels were significantly increased with increases in albuminuria (*p* = 0.02)	Furukawa et al. [53]
DGF	Plasma MDA	Prospective	40 transplant recipients	MDA levels on day 7 were independent predictors of 1-year graft function (*p* = 0.003)	Fonseca et al. [54]

**Table 3:** Differential expression of OS markers in CKD patients and healthy controls.

Molecule	Study Population	Mean Value	Reference
plasma AOPPs	62 type 2 diabetic patients/30 healthy controls	microalbuminuric DM: 252, DM: 155, controls: 124 (μmol/L)	Conti et al. [49]
plasma AOPPs	56 hypertensive patients/30 healthy controls	HT: 175, CKD for HT: 217, controls: 124 (μmol/L)	Conti et al. [49]
MN cells MDA	155 CKD patients (stages 3–5)/45 healthy controls	controls: 0.11, stage 3A: 0.57, stage 3B: 0.74, stage 4: 0.92, stage 5: 0.96 (nmol/mg protein)	Tomás-Simó et al. [52]
serum MDA-LDL	402 type 2 diabetic patients categorized according to eGFR and albuminuria levels	UAER (mg/d) < 30: 103, UAER 30–300: 109, UAER > 300: 135, eGFR > 90: 108, eGFR 60–90: 197, eGFR 60–30: 100, eGFR < 30: 141 (U/L)	Furukawa et al. [53]
plasma MDA	20 HD patients/16 CAPD patients/20 healthy controls	controls: 0.72, pre-HD: 0.83, post-HD: 1.39, CAPD: 1.26 (nmol/mL)	Ozden et al. [104]
plasma AOPPs	205 CKD stage 2–5 patients/40 controls	control: 24.9, stage 2: 38.5, stage 3: 50.5, stage 4: 58.4, stage 5: 68.2, stage 5D: 78.8 (μmol/L)	Azouaou Toualbi et al. [40]
plasma AOPPs	79 HD patients	total subjects: 149 (μmol/L)	Drüeke et al. [42]
plasma AOPPs	48 PD patients	baseline: 76.6, 1 year: 95.2 (μmol/L)	Gonzalez et al. [43]
plasma nitrite	351 regular blood donors/20 healthy individuals	controls: 351, one CVD risk factor (RF): 261, two CVD RFs: 253, three CVD RFs: 222, four CVD RFs: 171 (nmol/L)	Kleinbongard et al. [45]
apoB100-MDA	39 PD patients/40 healthy controls/61 HD patients	control: 14.4, PD: 17.11, HD: 15.68 (pmolMDA/mg apoB100)	Papadea et al. [105]
serum MDA-LDL	155 HD patients: 68 AS sufferers, 87 controls	AS sufferers: 120.63, controls: 72.65 (mg/dL)	Hou et al. [46]
serum MDA-LDL	100 HD patients: 52 PAS sufferers, 48 controls	PAS group: 119.67, controls: 78.38 (mg/dL)	Liu et al. [47]

## Abbreviations

ACE	Angiotensin-converting enzyme
ADMA	Asymmetric dimethylarginine
AGEs	Advanced glycation end products
AKI	Acute kidney injury
AOPPs	Advanced oxidation protein products
AOPPs-RSA	Advanced oxidation protein product-modified rat serum albumin
AS	Arterial stiffness
baPWV	Brachial–ankle pulse wave velocity
BMI	Body mass index
BP	Blood pressure
CAC	Coronary artery calcification
CAPD	Continuous ambulatory peritoneal dialysis
CAT	Catalase
cDBP	Central diastolic blood pressure
cfPWV	Carotid–femoral pulse wave velocity
CIN	Contrast-induced nephropathy
CKD	Chronic kidney disease
cNOS	Constitutive nitric oxide synthase
CRP	C-reactive protein
cSBP	Central systolic blood pressure
CV	Cardiovascular
CVD	Cardiovascular disease
DGF	Delayed graft function
DM	Diabetes mellitus
DN	Diabetic nephropathy
DTAC	Dietary total antioxidant capacity
EC-SOD	Extracellular superoxide dismutase
eGFR	Estimated glomerular filtration rate
eNOS	Endothelial nitric oxide synthase
EPO	Erythropoietin
ESKD	End-stage kidney disease
FGF23	Fibroblast growth factor 23
FMD	Flow-mediated dilation
HbA1c	Glycated hemoglobin
HD	Hemodialysis
HDL	High-density lipoprotein
HMA	Human mercaptoalbumin
HNA	Human non-mercaptoalbumin
HNE	4-hydroxy-2-nonenal
hOGG1	Human 8-oxoguanine DNA glycosylase 1
HPLC	High-performance liquid chromatography
HPMCs	Human peritoneal mesothelial cells
HSA	Human serum albumin
HT	Hypertension
ICAM	Intercellular adhesion molecule
IL-	Interleukin
IMA	Ischemia-modified albumin
IMT	Intima media thickness
LDL	Low-density lipoprotein
MDA	Malondialdehyde
MNC	Mononuclear cells
NAC	N-acetylcysteine
NADPH	Nicotinamide adenine dinucleotide phosphate hydrogen
NFκB	Nuclear factor κΒ
nNOS	Neuronal nitric oxide synthase
NO	Nitric oxide
NOS	Nitric oxide synthase
NT-proBNP	N-terminal pro-B-type natriuretic peptide
OS	Oxidative stress
Ox-LDL	Oxidized low-density lipoprotein
PAS	Peripheral arterial stiffness
PD	Peritoneal dialysis
pDBP	Peripheral diastolic blood pressure
PET	Peritoneal equilibration test
PMCs	Peritoneal mesothelial cells
Post-Tx	Post-transplantation
pSBP	Peripheral systolic blood pressure
PWV	Pulse wave velocity
RAAS	Renin–angiotensin–aldosterone system
RBC	Red blood cell
RCT	Randomized controlled trial
ROC	Receiver operating characteristic
ROS	Reactive oxygen species
sICAM	Soluble intercellular adhesion molecule
SOD	Superoxide dismutase
SSNS	Teroid-sensitive nephrotic syndrome
TAC	Total antioxidant capacity
TBARS	Thiobarbituric acid-reactive substances
TGF-β	Tumor growth factor β
TNF-α	Tumor necrosis factor α
UACR	Urine albumin–creatinine ratio
UAER	Urinary albumin excretion rate
VC	Vascular calcification
VCAM	Vascular cell adhesion molecule
VECM	Vitamin E-coated membrane
XDH	Xanthine dehydrogenase
XO	Xanthine oxidase
XOR	Xanthine oxidoreductase
